# Clinical and kinomic analysis identifies peripheral blood mononuclear cells as a potential pharmacodynamic biomarker in metastatic renal cell carcinoma patients treated with sunitinib

**DOI:** 10.18632/oncotarget.11686

**Published:** 2016-08-29

**Authors:** Gaёlle Noé, Audrey Bellesoeur, Audrey Thomas-Schoemann, Savithri Rangarajan, Faris Naji, Alicja Puszkiel, Olivier Huillard, Nathaniel Saidu, Lisa Golmard, Jerome Alexandre, Francois Goldwasser, Benoit Blanchet, Michel Vidal

**Affiliations:** ^1^ Assistance Publique Hôpitaux de Paris, Hôpital Cochin, UF Pharmacocinétique et Pharmacochimie, Paris, France; ^2^ UMR8638 CNRS, Faculté de Pharmacie, Université Paris Descartes, PRES Sorbonne Paris Cité, Paris, France; ^3^ Assistance Publique Hôpitaux de Paris, Hôpital Cochin, Service de Cancérologie Médicale, Paris, France; ^4^ PamGene International BV, ‘s-Hertogenbosch, The Netherlands; ^5^ Institut Curie, Département de Biopathologie, Paris, France; ^6^ U1016 INSERM, UMR 8104 CNRS, UMR-S1016, CARPEM, Université Paris Descartes, Sorbonne Paris Cité, Paris, France

**Keywords:** sunitinib, kinome, PBMC, renal carcinoma

## Abstract

**Background:**

Sunitinib is a protein tyrosine kinase (PTK) inhibitor that has immune-modulating properties. In this context, peripheral blood mononuclear cells (PBMC), mainly constituted by lymphocytes, could be a perfect surrogate tissue for identifying and assaying pharmacodynamic biomarkers of sunitinib. In this study, we investigated the changes in lymphocytes count as pharmacodynamic biomarker in metastatic renal cell carcinoma (mRCC) patients under sunitinib therapy. Thereafter, we studied the *ex vivo* effect of sunitinib and SU12262 (active metabolite) on PBMC from naïve mRCC patients using a high throughput kinomic profiling method.

**Methods:**

The prognostic value of total lymphocytes count between Day 0 and Day 21 (expressed as a ratio D21/D0) was retrospectively investigated in 88 mRCC patients under sunitinib therapy. PTK PamChip^®^ microarrays were used to explore prospectively the *ex vivo* effect of sunitinib and SU12662 on PTK activity in PBMC from 21 naïve mRCC patients.

**Results:**

In this retrospective study, D21/D0 lymphocytes ratio (Hazard Ratio, 1.83; CI95%, 1.24-2.71; p=0.0023) was independently associated with PFS. Interestingly, kinomic analysis showed that D21/D0 lymphocytes ratio and Heng prognostic model was statistically associated with the *ex vivo* sunitinib and SU12662 effect in PBMC.

**Conclusion:**

The present study highlights that D21/D0 total lymphocytes ratio could be a promising pharmacodynamic biomarker in mRCC patients treated with sunitinib. Additionally, it paves the way to investigate the kinomic profile in PBMC as a prognostic factor in a larger cohort of mRCC patients under sunitinib therapy.

## INTRODUCTION

Renal cell carcinoma (RCC) is the third most common malignancy of the urinary tract and is responsible each year for 338,000 new cases worldwide [1]. This cancer is considered to be an immunogenic tumor and a number of immunotherapeutic approaches have been exploited so far [2]. Until 2006, cytokines based immunotherapy (interferon-α and interleukin-2) was the only proven systemic therapy with a limited clinical efficacy. A better understanding of RCC biology has led to the approval of new targeted therapies, including kinase inhibitors such as vascular endothelial growth factor receptor (VEGF-R) and mammalian target of rapamycin (mTOR) inhibitors.

The use of kinase array technology to evaluate global kinase activities implied in cell signaling (« kinome ») in tumors from patients and to seek biomarkers for kinase inhibitors [3–7] is currently spreading. Recently, Anderson *et al.* have showed that the kinase profiling of clear cell RCC tumors could provide a functional classification strategy before starting a kinase inhibitor therapy [8]. Although these results are very promising, this strategy to process biopsy tumor before treatment initiation may fail to reflect current tumor dynamics and drug sensitivity, which may change during therapy. Additionally, processing repeated tumor biopsy cannot routinely be performed over the treatment course because of the invasive characteristic of the technique. Circulating cells could be an ideal biological matrix for biomarker discovery. In this context, we hypothesized that peripheral blood mononuclear cells (PBMC) could be a perfect surrogate tissue for identifying and assaying pharmacodynamic biomarkers of kinase inhibitors, as this tissue is readily accessible for repeat sampling throughout therapy. To the best of our knowledge, no data is currently available about the use of PBMC as surrogate tissue for the evaluation of global kinase activity in cancer patients.

Sunitinib, the first tyrosine kinase inhibitor developed for the VEGF pathway, is currently the standard first-line treatment for metastatic RCC (mRCC). Sunitinib is known to have immunomodulating properties in addition to its antiangiogenic activity [2, 9]. Interestingly a study by Powles *et al*. reported a significant reduction in the number of peripheral total lymphocytes (17%), CD3 (21%), CD4 (27%) and CD8 (29%) T-cells after completion of the first 6-week cycle of therapy in 43 mRCC patients [10]. No relationship was however found between this decline in lymphocytes count and progression free survival (PFS). Since each cycle consisted of 4 weeks of sunitinib followed by 2 weeks off the drug, it is possible that the effects on cell counts may be more marked before temporary discontinuation of sunitinib rather than at the end of the 6-week cycle. Furthermore, Adotevi *et al*. showed that the overall survival is significantly longer in mRCC patients showing a decrease in the number of Foxp3+ regulatory T-cells after 2 or 3 cycles of treatment [11]. In this context, further investigations are required to assess the changes in lymphocytes count as pharmacodynamic biomarker. Given PBMC are mainly constituted by lymphocytes (∼85%) [12], those investigations are a mandatory step before the evaluation of PBMC as surrogate tissue for kinomic analysis.

The present study aimed first, to retrospectively evaluate the changes in lymphocytes count at day 21 after sunitinib start in mRCC patients as a pharmacodynamic biomarker, and secondly, to assess the global protein tyrosine kinase (PTK) activity in PBMC from naïve mRCC patients before sunitinib treatment. A high throughput kinomic profiling method was employed to examine the effects of sunitinib and its active metabolite SU12662 on intracellular signaling pathways in PBMC.

## RESULTS

### Retrospective preliminary study

Eighty-eight mRCC patients treated with sunitinib were included. Baseline patients’ characteristics are summarized in Table 1. Median age was 62 years old [54-68]. The most frequent histological tumor type was clear cell carcinoma (71%). According to the Heng prognostic index model, most of the patients had an intermediate prognosis (57%) and about a third (36%) had a poor prognosis. Twenty-three patients (26%) developed lymphopenia during the first cycle of sunitinib treatment, with seven (8%) of grade ≥ 2.

**Table 1:** Baseline characteristics of the retrospective cohort

Characteristics	*N*= 88
**Demographic data**	
Sex, n (%)	
Male	62 (71)
Female	26 (29)
Age (years)	62 [54-68]
Age > 70 years-old, n (%)	18 (20)
BMI (kg/m^2^)	24.4 [22.3-27.9]
Lean Body Mass (kg)	56.0 [46.1-62.2]
ECOG performance status, n (%)	
0	19 (22)
1	50 (58)
2	12 (13)
3-4	7 (7)
**Renal cell carcinoma characteristics**	
Histological tumor type, n (%)	
Clear cell carcinoma	63 (71)
Papillary carcinoma	5 (6)
Other	20 (23)
Metastasis, n (%)	
Synchronous	43 (49)
Metachronous	45 (51)
Fuhrman's grade, n (%)	
1-2	13 (23)
3	28 (48)
4	17 (29)
Missing	30 (34)
Nephrectomy, n (%)	
Yes	76 (86)
No	12 (14)
Heng score, n (%)	
Favorable	6 (7)
Intermediate	50 (57)
Poor	32 (36)
**Baseline Biological data**	
Haemoglobin (g/dL)	12.6 [11.3-13.9]
Platelets (x10^9^/L)	280 [230-375]
Lymphocytes (x10^9^/L)	1.54 [1.13-1.99]
Neutrophils (x10^9^/L)	5.24 [3.69-6.83]
Lymphopenia before Sunitinib treatment, n (%)	36 (41)
LDH	
Increased above ULN	22 (29)
Normal	54 (61)
Missing	12 (14)

BMI, body mass index; ECOG, Eastern Cooperative Oncology Group; LDH, lactate dehydrogenase; ULN, Upper Limit of Normal.

Results are expressed as median [interquartile range] or frequency (percent).

The median PFS in the cohort was 234 days (confidence interval, CI95%, 179-289). In univariate analysis (Table 2), the lymphocytes ratio between day 0 (was the single hematological ratio at day 21 associated with PFS. An increase in lymphocyte count on day 21 was significantly associated with a poorer prognosis (*p* = 0.0028). Multivariate Cox proportional hazards model analysis showed that lymphocyte D21/D0 ratio (hazard Ratio [HR], 1.83; CI95%, 1.24-2.71; *p*= 0.0023), ECOG (performance status 0-1 (HR, 0.44; CI95%, 0.23-0.84; *p* = 0.0135) and body mass index (HR, 0.86; CI95%, 0.80-0.93; *p* = 0.0001) were independently associated with PFS.

**Table 2:** Results of Univariate and Multivariate Analysis of Progression Free Survival prognostic factors (n=88)

Variables (units)	Univariate analysis		Multivariate analysis	
	Hazard Ratio [CI95%]	*p*-value	Hazard Ratio [CI95%]	*p*-value
Male Sex	0.71 [0.42-1.18]	0.18		
Age (years)	0.99 [0.97-1.02]	0.58		
ECOG 0-1	0.56 [0.31-1.01]	0.053	0.44 [0.23-0.84]	0.0135
Lean body mass (kg)	0.96 [0.94-0.98]	0.0003		
BMI (kg/m^2^)	0.87 [0.82-0.93]	<0.0001	0.86 [0.80-0.93]	0.0001
Metachronous metastasis	0.71 [0.44-1.14]	0.154		
Clear cell carcinoma histological type	0.44 [0.26-0.75]	0.0025		
Fuhrman's grade 4*	2.20 [1.15-4.19]	0.0167		
Nephrectomy	0.31 [0.15-0.62]	0.0010		
Heng score		0.0216		
Favourable (n=6)	0.33 [0.12-0.97]			
Intermediate (n=50)	0.54 [0.33-0.89]			
Poor (n=32)	1			
Increased LDH**	1.21 [0.70-2.10]	0.495		
Steroïds comedication	1.22 [0.62-2.39]	0.569		
Baseline Lymphocytes (x10^6^/L)	0.77 [0.58-1.02]	0.0649		
Baseline NLR	1.05 [1.00-1.11]	0.041		
Haemoglobin D21/D0	1.19 [0.13-10.95]	0.877		
Platelets D21/D0	0.96 [0.43-2.12]	0.909		
Lymphocytes D21/D0	1.72 [1.21-2.45]	0.0028	1.83 [1.24-2.71]	0.0023
Neutrophils D21/D0	1.50 [0.46-4.84]	0.5		
G3-4 induced-lymphopenia during the first cycle	1.23 [0.53-2.87]	0.625		
No lymphopenia prior Sunitinib treatment	1.09 [0.67-1.79]	0.73		
Composite AUC_ss_^a^ at D21	0.93 [0.69-1.25]	0.621		

AUC_ss_, Area under the curve of plasma concentration at steady-state; BMI, body mass index; CI95%, confidence interval 95%; ECOG, Eastern Cooperative Oncology Group; G, Grade according to NIC-CTCAE V4; LDH, Lactate Dehydrogenase; NLR, Neutrophil to Lymphocyte Ratio; PFS, progression-free survival;

* n=58

** n=76

a Composite AUC_ss_ is the sum of sunitinib and SU12662 (active metabolite) AUC. It was assayed at day 21 after the sunitinib start.

### Basal PTK activity in PBMC from mRCC patients and healthy volunteers

In continuum of the previous clinical results, we conducted a study to compare the PTK activities in PBMC from mRCC patients and healthy volunteers using peptide microarrays. The clinical and biological characteristics of naïve mRCC patients population (*n* = 21) before sunitinib treatment initiation are summarized in Table 3. Median age was 50 [48-57] years in healthy volunteers (*n* = 12), and half of them were male.

**Table 3:** Baseline characteristics of patients included in the kinomic analysis

Baseline characteristics	Patients (*n*=21)
**Demographic data**	
Sex, n (%)	
Female	7 (33)
Male	14 (67)
Age (years)	69 [60-74]
BMI (kg/m^2^)	25.0 [24.1-27.0]
ECOG, n (%)	
0	4 (19)
1	9 (43)
2	7 (33)
3	1 (5)
**Renal cell carcinoma characteristics, n (%)**	
Histological tumor type, n (%)	
Clear cell carcinoma	17 (77)
Papillary carcinoma	1 (9)
Other	3 (14)
Metastasis	
Yes	21 (100)
No	0 (0)
Heng score, n (%)	
Favorable	2 (10)
Intermediate	12 (57)
Poor	7 (33)
Prior nephrectomy, n (%)	
Yes	17 (81)
No	4 (19)
**Lymphocytes profile at day 0**	
Baseline total lymphocytes (x10^9^/L)	1.3 [1.2-1.7]
Lymphopenia before Sunitinib treatment, n (%)	13 (62)

BMI, body mass index; ECOG, Eastern Cooperative Oncology Group;

Results are expressed as median [interquartile interval] or frequency (percent)

**Figure 1:**
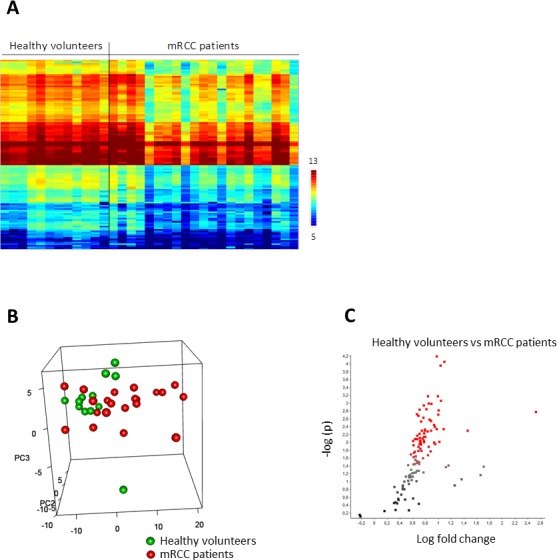
Basal protein tyrosine kinase activity profiles (Log2Signal) in PBMC from healthy volunteers (*n* = 12) and metastatic renal cell carcinoma patients (*n* = 21) **A.** The heatmap shows rows (representing 110 peptides of the “QC List”) sorted by hierarchical clustering using Euclidean distance metrics and complete linkage. **B.** The 3D plot shows 3 new variables (PC1-3) obtained after applying Principal Component Analysis (PCA), each point representing a sample colored according to PBMC source. **C.** The volcano plot shows the result of *T*-test: the effect size, i.e. LFC (Log_2_Signal (Healthy) - Log_2_Signal (mRCC)), versus significance (−Log_10_p). Peptides with significant p-values (< 0.05) are marked in red.

A large interindividual variability in kinomic profiles (Figure 1A) was observed, especially in mRCC patients, which could not be correlated with various baseline clinical and biological parameters tested. Even so, unsupervised PCA) showed a tendency of separation between patients and healthy volunteers (Figure 1B) with a tendency of clustering in the case of healthy volunteers. Furthermore, several peptides showed significantly lower phosphorylation levels in PBMC from patients when compared to healthy volunteers (76 with *p* < 0.05, 45 with *p* < 0.01) (Figure 1C), suggesting that overall kinomic profiles of naïve mRCC patients’ PBMC could be differentiated from those of healthy individuals. Functional ontology enrichment analysis using Metacore software (GeneGO)^TM^ showed that several pathways were deregulated in patient PBMC, including immune response (FC epsilon RI-, CD28-, CCR5-, CD16- signaling), the EGFR (ERK inhibition, PI3K/AKT- and MAPK- signaling), FGF-family and FGFR-signaling, along with chemotaxis (CXCR4- signaling).

### *Ex vivo* PTK inhibitory effects of sunitinib and SU12662

**Figure 2:**
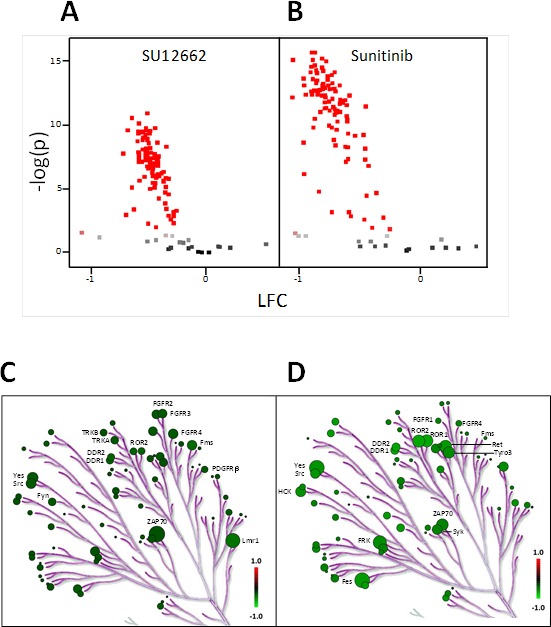
*Ex vivo* inhibition of sunitinib and SU12662 on protein tyrosine kinase activity in PBMC from metastatic renal cell carcinoma patients (*n* = 21) **A.** and **B.** : The volcano plots show the result of an ANOVA post-hoc test. The effect size i.e. LFC (Log_2_Signal (SU12662 **A.** OR Sunitinib **B.**) - Log_2_Signal (DMSO)) *versus* significance (−Log_10_P). Peptides with significant p-values (< 0.05) are marked in red. BioNavigator® interfaced with R was used to generate the graphs. **C.** and **D.** : Upstream kinase analysis of the same data set using BioNavigator® interfaced with R, were mapped to a phylogenetic tree of the kinome using the Kinome Renderer tool [35]. Only the 15 main kinases inhibited by sunitinib or SU12662 are presented. The size of the circles indicates the specificity score of the corresponding kinases and the green color relates to the effect size with lower inhibition being darker. Image reproduced courtesy of Cell Signaling Technologies Inc.

The next step was to study the *ex vivo* effect of sunitinib or SU12662 (active metabolite) in PBMC lysates from 21 naïve mRCC patients. Inhibition profiles obtained after *ex vivo* exposure to either sunitinib or SU12662 showed that PTK substrate phosphorylation levels were reduced. Interestingly, the inhibitory effect of sunitinib was stronger than that of SU12662. (Figure 2A, 2B) allowed the identification of peptides that were significantly inhibited (106 and 102 peptides for sunitinib and SU12662, respectively, *p* < 0.05). Putative upstream PTK were determined by using Kinexus Kinase Predictor. The top 15 PTK that were inhibited are presented on kinome trees for sunitinib (Figure 2C) and SU12662 (Figure 2D). Amongst them, were some that are implicated in immune response, in particular Zap70, Src, Fyn, Yes1 and DDR1.

### Correlation of *ex vivo* inhibition profiles and heng prognostic score

**Figure 3:**
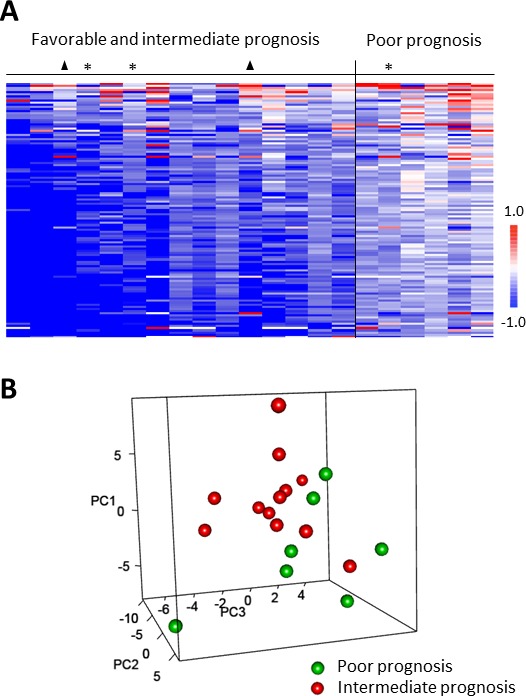
Correlation of *ex vivo* sunitinib-related inhibition profiles in PBMC from metastatic renal cell carcinoma patients to Heng prognostic scores **A.** The heatmap of LFC (Log_2_Signal (Sunitinib) - Log_2_Signal (DMSO)) shows columns (*n* = 21) sorted by column mean and rows (representing 110 peptides of the “QC List”) sorted by row mean. When Heng prognostic score was overlaid on the data, higher inhibition (lower LFC) corresponded to favorable (▲) or intermediate scores except for 3 outliers (*). **B.** The 3D plot shows 3 new variables (PC 1-3) obtained after applying Principal Component Analysis (PCA), each point (*n* = 19) representing a sample colored according to Heng prognostic score. Only intermediate and poor prognostic groups were included in this statistical analysis.

The *ex vivo* inhibition profiles of sunitinib and SU12662 were statistically correlated with Heng prognostic scores. Thus, mRCC patients with poor prognosis presented a lower inhibitory effect of either sunitinib (Figure 3A) or SU12662 (Supplementary Figure 1A). A tendency of separation between intermediate and poor prognosis groups was observed after applying PCA, and phosphorylation inhibition was significantly different between these 2 prognosis groups (53 and 23 peptides for sunitinib and SU12662, respectively, *p* < 0. 05) (Figure 3B and Supplementary Figure 1B). Using GeneGO^TM^, we observed that signaling pathways including VEGF-, HGF-, FGFR- and EGFR- for sunitinib and PDGFR for SU12662 were statistically more inhibited in the intermediate prognosis group. Kinases involved in the immune response such as, Itk, Tec, Btk, Lyn, Syk and Zap 70 were the most inhibited by both sunitinib and SU12662 in the intermediate group.

### Relationship between *ex vivo* inhibition profiles and lymphocytes D21/D0 ratio

**Figure 4:**
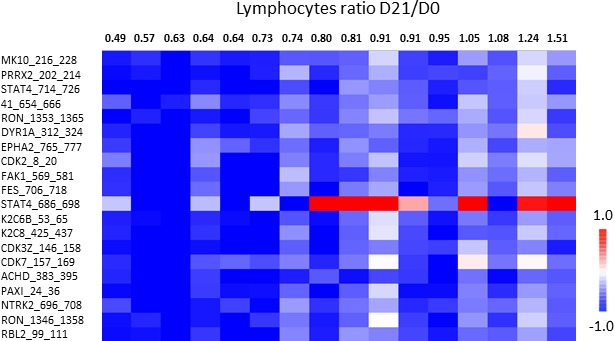
Relationship between *ex vivo* sunitinib-related inhibition profiles in PBMC from metastatic renal cell carcinoma patients and lymphocytes ratio D21/D0 (*n* = 16) The heatmap of LFC (Log_2_Signal (Sunitinib) - Log_2_Signal (DMSO)) shows 20 peptides (Y axis) which present linear correlation between LFC and lymphocytes ratio (X axis). Each column represents one patient. The rows show 20 significant (*p* < 0.05) peptides derived from a one-way ANOVA. When lymphocytes ratio was overlaid on the data, higher inhibition (lower LFC) corresponded to lymphocytes count decreased at D21. Graph on the right show the same results for one peptide in a dot plot.

Based on the prognostic value of D21/D0 lymphocytes ratio, we investigated the relationship between this ratio and the *ex vivo* kinomic inhibition profiles sunitinib and SU12662. Five patients could not be assessed on day 21 due to death (*n* = 1) and early discontinuation of treatment related to severe toxicities (*n* = 4). Interestingly, a linear relationship was found with both the *ex vivo* inhibition profiles of sunitinib (20 peptides with *p* < 0.05) and SU12662 (18 peptides with *p* < 0.05). Lower D21/D0 lymphocytes ratio was associated with higher phosphorylation inhibition of these peptides for sunitinib (Figure 4) and SU12662 (Supplementary Figure 2).

## DISCUSSION

This retrospective clinical study is the first to show that D21/D0 lymphocytes ratio could be used as a pharmacodynamic biomarker in mRCC patients under sunitinib therapy. The kinomic study that followed shows that both sunitinib and SU12662 induce PTK inhibition in PBMC. Additionally, their *ex vivo* effect on PBMC are statistically correlated with both Heng prognostic score and D21/D0 lymphocytes ratio. Together, these data suggest that a stronger *ex vivo* sunitinib- and SU12662-induced inhibition in PBMC kinome profile before sunitinib start would be associated with a better prognosis in mRCC patients.

The present retrospective clinical study highlights that a decrease in lymphocytes count on D21 was associated with a longer PFS and could therefore be an interesting prognostic factor. Pretreatment lymphopenia is traditionally considered as a poor prognostic factor in naïve RCC patients [13]. The D21/D0 lymphocytes ratio is not necessarily associated with pathological values and constitutes a dynamic parameter reflecting rapid variations in lymphocytes count under sunitinib treatment. Moreover, this ratio seems to be a better prognostic factor than either pre-treatment lymphopenia or neutrophil to lymphocyte ratio (NLR). Different clinical studies have reported that lower baseline NLR is associated with longer PFS, suggesting baseline NLR as an interesting prognostic factor [14–16]. In the present study, lower baseline NLR was only associated with longer PFS in the univariate analysis, probably because all the patients included in our cohort were treated in first line with sunitinib and could therefore have a less inflammatory disease than other patients included in the previous studies. Additionally, the baseline NLR ratio indicative of the balance between host immunity and cancer-related inflammation does not take account of the interindividual variability in the pharmacodynamic effect of sunitinib, while the total peripheral lymphocytes D21/D0 ratio does. The decline in lymphocytes count on D21 seems unlikely due to a cytotoxic drug effect on peripheral lymphocytes for two reasons. Firstly, it was associated with an increased PFS in our cohort. Secondly, Krusch *et al*. did not report *in vitro* cytotoxicity of increased sunitinib concentrations on PBMC [17]. By contrast, two recent studies have documented that sunitinib enhances T-lymphocytes recruitment in tumor microenvironment [18, 19]. Thus, the sunitinib-related inhibition of VEGF signaling results in up-regulation of chemokines CXCL10 and CXCL11 (chemoattractant for T-cells) in tumor vessels [19]. Additionally, sunitinib is known to reduce intratumoral content of myeloid-derived suppressor cells in human renal cell carcinoma which can also contribute to increase T-cells there [20]. Altogether, these recent findings suggest that the association between a decrease in lymphocytes count on D21 and a longer PFS could be related to a change in the compartmental distribution of lymphocytes, characterized by a progressive infiltration in tumor tissue and therefore, a better anti-tumor immunity. Further investigations are however warranted to support this hypothesis.

To the best of our knowledge, the present study is the first to show that kinomic profiles of PBMC from naïve mRCC patients would be different from healthy volunteers. Twine *et al.* have however reported disease-associated differences in transcriptome profiles of PBMC from RCC patients compared to PBMC of healthy volunteers [21]. They observed heterogeneity in the expression of these transcripts across RCC patients. Interestingly, we found that the activity of most PTK was lower in PBMC from naïve mRCC patients than those in healthy volunteers. Immune dysfunction has been well documented in RCC patients, especially with the tumor infiltrating T-cells anergy [22]. T-cells exhaustion is known to be related in part to the expression of inhibitory molecules such as Programmed Death 1 (PD-1), whose level of expression is enhanced by VEGF-A [23, 24]. A recent study showed that the plasma level of VEGF-A was 3- to 4-fold higher in mRCC patients than in healthy volunteers [25]. Besides, another study documented an increased PD-1 expression on circulating T-cells, NK cells and monocytes from RCC patients [26]. Taken together, these results suggest that the lower PTK activity observed in our mRCC cohort could be related to an increased expression of PD-1 on PBMC. Another explanation may be related to the difference in age between mRCC patients and healthy volunteers (69 *vs* 50 years old). Indeed, intrinsic “senescence” is known to compromise the activation pathways in lymphocytes from the elderly [27]. Additionally, an increase in the number of PD-1-expressing T-lymphocytes has been documented in aged mice, which confers to these cells an exhausted phenotype [28]. These different factors could also contribute to the lower PTK activity in our mRCC cohort.

The present study is the first to provide kinome trees of sunitinib and its active metabolite SU12662 for PTK in PBMC from naïve mRCC patients. As expected, our results showed that sunitinib and SU12662 inhibited many PTK. In literature, well-known targets for sunitinib are Flt-1 (VEGFR-1), KDR (VEGFR-2), PDFGR, kit, Flt-3 and RET [29]. Even though these targets were not the most inhibited in our study, their inhibition validates our approach of using PBMC as a model system. Discrepancies between our sunitinib kinome tree and the documented tree for sunitinib [30] could be explained by the latter being built from the *in vitro* data, which represents sunitinib affinity for purified kinases. Additionally, it does not take into account the PTK expression level in cells. Finally, the present study highlights for the first time a stronger inhibitory effect of sunitinib than SU12662 on shared targets like Zap70, some Src family kinases (Src, Fyn, Yes1) and DDR1, which are respectively involved in lymphocytes activation [31], lymphocytes development [32] and T-cells migration [33]. These results could constitute a first step to decipher these mechanisms in more depth.

The large interindividual variability in sensitivity towards both sunitinib and SU12662 could contribute to the variability in immunomodulatory effects in mRCC patients. Among the different baseline parameters tested, the Heng prognostic score was the single factor of variability identified. Interestingly, sunitinib and SU12662 exhibited less *ex vivo* inhibitory effects in PBMC from mRCC patients with a poor Heng prognostic score. From clinical data, the recent ESMO clinical practice guidelines recommend the use of mTOR inhibitor in first-line treatment of mRCC patients with poor prognosis, while sunitinib remains the golden standard in other patients [34]. Taken together, these results suggest that sunitinib should be less efficient in mRCC patients exhibiting a lower *ex vivo* inhibitory effects of either sunitinib or SU12662 in their PBMC. Additionally, the retrospective study highlights the prognostic value of lymphocytes D21/D0 ratio. Finally, the kinomic analysis has identified a positive relationship between the D21/D0 lymphocytes ratio and the *ex vivo* level inhibition by sunitinib (or SU12662) for some peptides. In this context, these preliminary results pave the way to investigate the kinomic profile in PBMC as a prognostic factor in a larger cohort of mRCC patients under sunitinib therapy.

In conclusion, the present study highlights that D21/D0 total lymphocytes ratio could be a promising pharmacodynamic biomarker in mRCC patients treated with sunitinib in first line. Additionally, our data suggest that the kinomic analysis in PBMC from mRCC patients could be a useful tool to identify good candidates for sunitinib treatment. Moreover, this first kinomic analysis in PBMC paves the way to seek mechanisms that drive tumor cell to immune escape in mRCC patients under sunitinib.

## MATERIALS AND METHODS

### Retrospective preliminary study

A retrospective study was conducted in mRCC patients treated with first-line sunitinib therapy from June 2006 to January 2015 in the oncology department of Cochin Hospital in Paris, France. From medical records, baseline clinical and biological parameters were collected. Biological parameters included hematological specifications (haemoglobin, leukocytes, neutrophils, lymphocytes and platelets) before treatment starts (D0) and on day 21 (D21) of the first cycle of treatment. Each one was expressed as a ratio (D21/D0) to reflect their global evolution besides pathological values. Finally, steady-state plasma composite (sunitinib + SU12662) exposure at D21 was recorded.

### Patient selection for kinomic profiling

From March 2015 to November 2015, a total of 21 naïve mRCC patients and 12 healthy volunteers were included for the kinomic analysis. The latter was in compliance with the Declaration of Helsinki and approved by the local medical ethical board (N° 316-12). All subjects gave their written informed consent to participate in the study.

### Sample collection and lysis

PBMC from healthy volunteers and patients (at D0) were isolated by density-gradient centrifugation in Ficoll. Cells (3.5 million) were lysed in 140 μl of the mammalian protein extraction (M-PER) reagent (Thermo Scientific, Courtaboeuf, France), supplemented with 1:100 Halt's phosphatase and protease inhibitors (Thermo Scientific) before centrifugation (12000 rpm, 15 min, 4°C). Aliquots of the supernatant were stored at -80°C. Protein quantification was performed using standard Bradford protein assay (Thermo Scientific). PBMC were used rather than lymphocytes to minimize the influence of FACS sorting on PTK activity.

### Kinomic profiling assay

PTK activity profiling was performed using a PamStation^®^12 (PamGene International B.V.,'s Hertogenbosch, The Netherlands) and PTK PamChip^®^4 with 4 identical arrays. On each array, 142 tyrosine-containing peptides (13 amino acids long), derived from known human phosphorylation sites, are immobilized.

An assay incubation mixture, containing 2 to 4 μg of protein (depending on total protein available), 100 μM ATP and fluorescein isothiocyanate (FITC) labeled antiphosphotyrosine antibody (PamGene), was added in each array. The mixture was pumped up and down through the porous membrane. Peptide phosphorylation was monitored during the incubation by taking images with a CCD camera in combination with Evolve software v. 1.2 (PamGene) allowing real-time recording of the reaction kinetics. After washing of the arrays, fluorescence was detected at different exposure times (20, 50, 100 and 200 ms).

Kinomic profiles of healthy volunteers and sunitinib-naïve mRCC patients at baseline (D0) were studied by spiking in vehicle (0.1% of dimethyl sulfoxide, DMSO) into the assay incubation mixture and performing the PTK kinomic assay. The *ex vivo* inhibitory effect of sunitinib and SU12662 in mRCC patient lysates was studied by spiking in sunitinib (1 μM), SU12662 (1 μM) or DMSO (0.1%) into the assay incubation mixture before performing the PTK kinomic assay.

### Statistical analysis

For the retrospective clinical study, descriptive statistics used median [interquartile interval] for quantitative variables and percentages for qualitative ones. Ratio D21/D0 for each hematological parameter was assessed to explore the pharmacodynamic effect of sunitinib on peripheral blood cells. PFS was analyzed in the framework of survival analysis. PFS was estimated with the Kaplan-Meïer method. Clinical and biological predictive factors were first assessed using univariate Cox models. Secondly, factors with a p-value lower than 0.10 were entered into a stepwise Cox model regression. The final multivariate Cox model contained the set of factors with a p-value lower than 0.05 by the Wald test. All computations were performed with the SAS V9.3 statistical package (SAS institute Inc., Cary, NC, USA).

For kinomic analysis, image analysis and signal quantification were performed using the BioNavigator^®^ software v. 6.1 (PamGene), whilst data analysis was done using the same software but interfaced with R (Bioconductor). Peptides that showed kinetics (increase in signal intensity in time) were preselected (“QC list”) and Log_2_-transformed (“Log_2_Signal”). Log fold change (LFC) was determined from *ex vivo i*nhibitor data (Log_2_Signal Inhibitor - Log_2_Signal DMSO). *Ex vivo* inhibitor effects were evaluated for each peptide using ANOVA. Kinexus Kinase Predictor was used to determine putative upstream PTK, which were mapped to a phylogenetic tree of the kinome using the Kinome Renderer tool. Per-peptide differences between Heng pronostic groups were evaluated using two-tailed *t*-tests and unsupervised multivariate clustering of samples was evaluated with Principal Component Analysis (PCA). For these analyses, data from patients with good prognosis (*n* = 2) were not included in the study as they lacked statistical power. A one-way ANOVA was used to compare the lymphocytes ratio D21/D0 to LFC signal intensity. MetaCore (GeneGo) ^TM^ (Thomson Reuters, U.S.A) was used for pathway and network analysis.

## SUPPLEMENTARY MATERIAL FIGURES


