# Supplementary material for: Clinical and kinomic analysis identifies peripheral blood mononuclear cells as a potential pharmacodynamic biomarker in metastatic renal cell carcinoma patients treated with sunitinib
Source: Oncotarget. 2016 Aug 29;7(41):67507–20. doi: 10.18632/oncotarget.11686 (PMC5341893; doi:10.18632/oncotarget.11686)
Supplement: Supplementary file 1 [file oncotarget-07-67507-s001.pdf]

Clinical and kinomic analysis identifies peripheral blood mononuclear cells as a potential pharmacodynamic biomarker in metastatic renal cell carcinoma patients treated with sunitinib

Supplementary Material

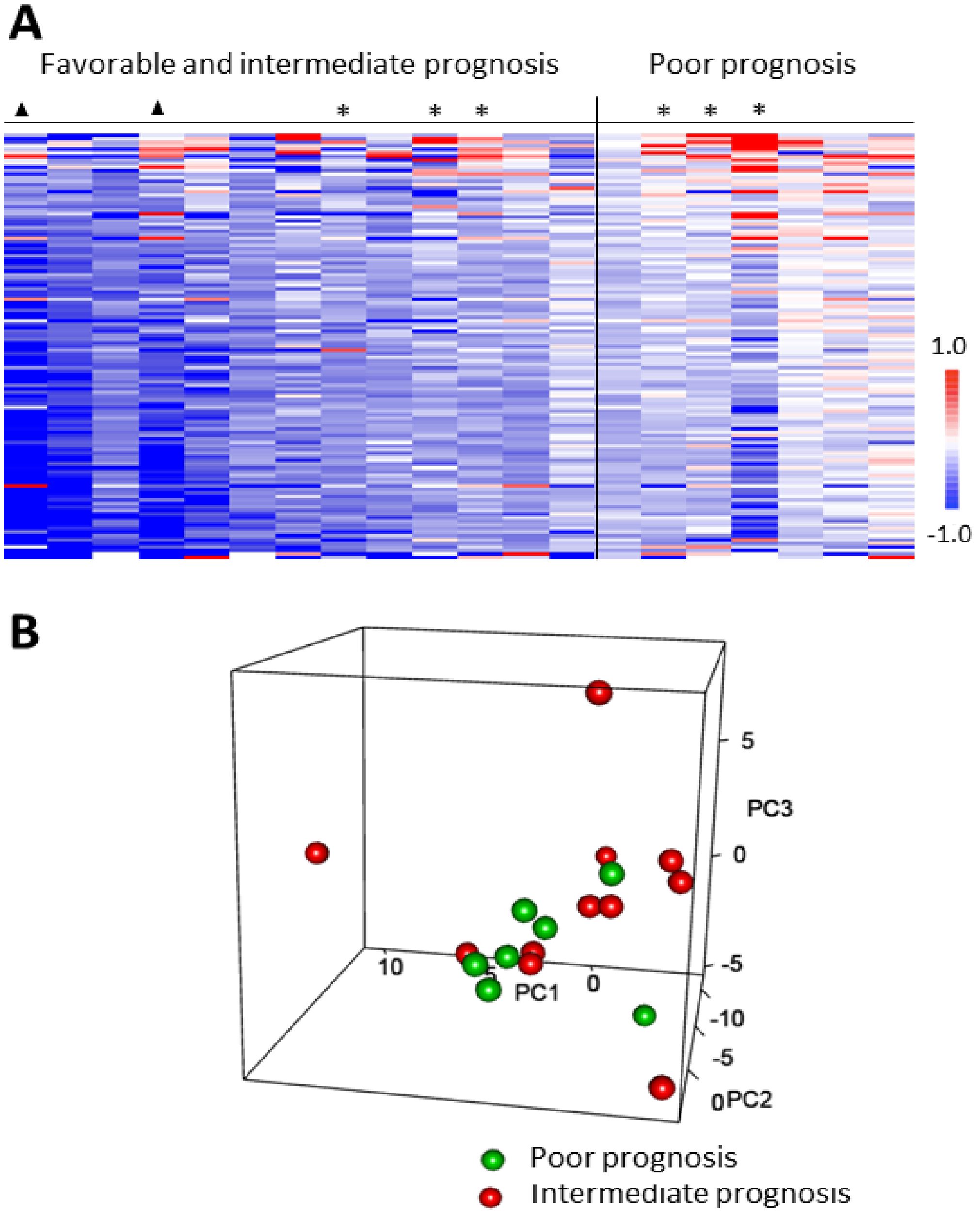

Supplementary Figure 1: Correlation of SU12662 *ex vivo* inhibition profiles in PBMC from mRCC patients Heng prognosis scores.

A. The heatmap of LFC (Log2Signal (SU12662) - Log2Signal (0.1% DMSO)) shows columns (n=20) sorted by column mean and rows (representing 110 peptides of the "QC List") sorted by row mean. When Heng prognosis score was overlaid on the data, higher inhibition (lower LFC) corresponded to favorable (▲) or intermediate scores except for 6 outliers (*).

B. The 3D plot shows 3 new variables (PC1-3) obtained after applying Principal Component Analysis (PCA), each point (n=18) representing a sample colored according to Heng prognosis score. Only intermediate and poor prognostic groups were included in this statistical analysis.

One patient could not be evaluated because of a technical issue.

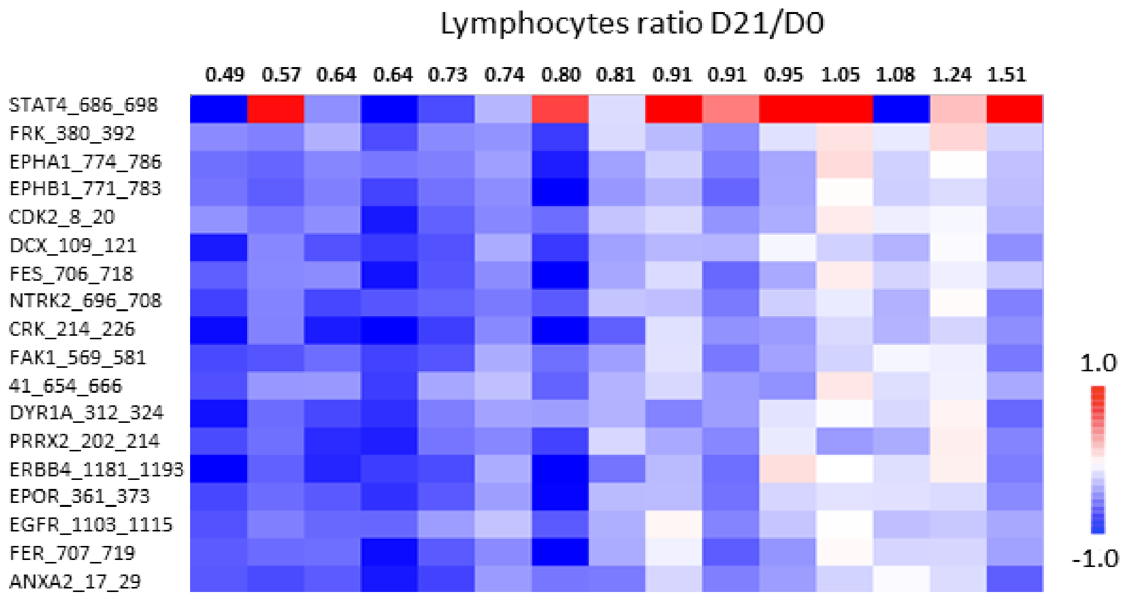

Supplementary Figure 2: Correlation of SU12662 *ex vivo* inhibition profiles in PBMC from mRCC patients (n=15) to lymphocytes ratio D21/D0.

The heatmap of LFC (Log2-signal (SU12662) - Log2-signal (DMSO)) shows 18 peptides (Y axis) which present linear correlation between LFC and lymphocytes ratio (X axis). Each column represents one patient. When lymphocytes ratio was overlaid on the data, higher inhibition (lower LFC) corresponded to lymphocytes count decreased at D21. One patient could not be evaluated with because of a technical issue.
